# Neurocognitive consequences of adolescent sleep disruptions and their relationship to psychosis vulnerability: a longitudinal cohort study

**DOI:** 10.1038/s44184-024-00058-x

**Published:** 2024-05-07

**Authors:** Julien Ouellet, Roxane Assaf, Mohammad H. Afzali, Sima Nourbakhsh, Stéphane Potvin, Patricia Conrod

**Affiliations:** 1 CHU Sainte-Justine Research Center, Montreal, QC Canada; 2 Department of Neuroscience, Université de Montréal, Montreal, QC Canada; 3 Department of Psychiatry, Université de Montréal, Montreal, QC Canada; 4 Institut Universitaire en Santé Mentale de Montréal Research Center, Montreal, QC Canada; 5 Department of Pediatrics, Université de Montréal, Montreal, QC Canada

**Keywords:** Cognitive neuroscience, Human behaviour

## Abstract

Adolescence is a key period for neurocognitive maturation where deviation from normal developmental trajectories may be tied to adverse mental health outcomes. Cognitive disruptions have been noted in populations at risk for psychosis and are known to accompany periods of sleep deprivation. This study aims to assess the role of cognition as a mediator between sleep disruptions and psychosis risk. A cohort of 3801 high school students (51% female, mean age = 12.8, SD = 0.45 years) was recruited from 31 Montreal high schools. Measures of sleep, psychotic-like experiences, inhibition, working memory, perceptual reasoning, and delayed recall were collected from participants on a yearly basis over the five years of their high school education. A multi-level model mediation analysis was performed controlling for sex and time squared. Response inhibition was shown to be associated with, and to mediate (*B* = −0.005, SD = 0.003, *p* = 0.005*) the relationship between sleep disruptions (*B* = −0.011, SD = 0.004, *p* < 0.001*) and psychotic-like experiences (*B* = 0.411, SD = 0.170, *p* = 0.005*). Spatial working memory deficits on a given year were associated with a higher frequency of psychotic-like experiences that same year (B = −0.046, SD = 0.018, *p* = 0.005*) and the following year (B = −0.051, SD = 0.023, *p* = 0.010*), but were not associated with sleep disturbances. No significant associations were found between our variables of interest and either delayed recall or perceptual reasoning at the within person level. Findings from this large longitudinal study provide evidence that the association between sleep disruptions and psychosis risk is specifically mediated by inhibitory rather than general cognitive impairments. The association of spatial working memory, response inhibition, and sleep disruptions with psychotic-like experiences suggests that these factors may represent potential targets for preventative interventions.

## Introduction

Adolescence is a critical developmental phase characterised by the fine-tuning of multiple cognitive skills^1^. The parallel development of skills such as inhibition and working memory is a predictor of many life outcomes with deviations from normal developmental trajectories being associated with various psychopathologies, especially in the context of thought disorders such as schizophrenia, schizoaffective disorder and psychotic bipolar disorder^2^. Comparatively mild cognitive deficits can be detected in the developmental and prodromal phases of the disease. These deficits peak during the first psychotic episode and a gradual cognitive deterioration is observed throughout the chronic phase of the disease^2^. Individuals considered at high risk of psychosis are shown to be a greater risk of transitioning if they suffer from cognitive impairments^3^. Overall, a general association between psychotic disorders and response inhibition has been corroborated by a variety of testing paradigms^4,5^. For instance, a recent study shows that patients with schizophrenia display worse accuracy and reaction time on a go/no-go task compared to healthy controls^6^. While a number of cognitive dimensions are disrupted in psychotic disorders, some models suggest that working memory and inhibitory control may represent core aetiological factors of the disease. For example, the Waters model posits that inhibitory deficits directly contribute to auditory hallucinations^7^. In addition, working memory deficits have also been found in individuals at risk of psychosis^8^, with some studies suggesting worse working memory performance may further increase the risk of a later conversion to psychosis in this population^8,9^. Other memory domains are also affected in psychosis, with delayed recall showing a tendency to deteriorate with increasing symptom severity. This results in more significant deficits being observed in individuals experiencing a first psychotic episode compared to those with ultra-high-risk mental states^10^. A similar deterioration in perceptual reasoning is also observed from late adolescence to the onset of a first psychotic episode^11^. Importantly, there are disagreements between studies as to which cognitive domains are first affected, most disrupted and most predictive.

### Sleep, psychosis and psychotic-like experiences

In recent years, new research findings have promoted sleep from a mere comorbidity of psychosis and schizophrenia to a potentially treatable causal target^12^. A study of 1809 participants with schizophrenia revealed that 50.1% had clinically significant levels of insomnia^13^. According to a recent meta-analysis, medication-free patients with psychosis display a higher sleep latency, lower overall sleep time, and decreased sleep efficiency^14^. Left untreated, co-morbid sleep disorders may lead to a significant increase in the positive and cognitive symptoms reported by these patients in addition to an overall decrease in their quality of life^15^. Conversely, actigraphic studies of adolescents at ultra-high-risk of psychosis revealed a marked increase in the frequency of wake after sleep onset and a decrease of sleep efficiency in this population^16^. A recent longitudinal study supports a temporal precedence of sleep on most psychotic symptoms with the notable exception of paranoid delusions for which the association with sleep appears bi-directional^17^.

In addition, a robust association has been found between sleep disruptions and the frequency of psychotic-like experiences (PLEs)^18^. Sleep disruptions are tied to a 4-fold increase in the risk of experiencing PLEs^19^, while PLEs are in turn associated with a 3.5-fold increase of a later conversion to psychosis^20^. In addition, PLEs are also considered a marker of risk for a variety of psychiatric disorders including major depressive, bipolar, generalised anxiety, post-traumatic stress, adult separation anxiety, eating and substance use disorders^21^.

### Sleep and cognition

Sleep is critical to mental health and optimal functioning across a broad range of cognitive domains^22,23^. The influence of sleep deprivation on cognition may be especially consequential during adolescence where it may interfere with a number of key neuromaturational processes^24^. One prospective study showed that poor sleep in childhood is associated with reduced inhibitory faculties in adolescence^25^. A meta-analysis concluded that adults with insomnia experience noticeable deficits in higher-level cognition especially in terms of working memory, episodic memory and problem solving abilities^26^.

One could make the argument that these cognitive disruptions are the underlying cause of sleep disturbances, as they could potentially interfere with sleep through irregular arousal or behavioural patterns. However, studies involving experimentally induced sleep restrictions have demonstrated comparable impacts on cognitive disturbances, which suggests that sleep loss itself is the issue^27^. Additionally, while attributes like inhibition could theoretically influence one’s ability to maintain proper sleep hygiene, empirical research actually indicates that sleep disruptions hinder inhibition, whereas the inverse doesn’t hold true^28^. While some facets of executive functions were thought to exert a positive effect on sleep by facilitating the maintenance of sleep hygiene, results have failed to show evidence of a reverse causal relationship between response inhibition and sleep difficulties^28^. This suggests that sleep disruptions affect cognition more than cognitive deficits affect sleep.

PLEs are a known predictor of psychosis^29^ and represent an opportunity to study at risk individuals before the onset of the disease. In this study, we test the role of cognition as a mediator between sleep parameters and PLEs using data from a longitudinal cohort study^30^. We hypothesise that poor sleep on a given year will interfere with cognitive functions and that worse cognitive performance will be associated with a concurrent increase in PLEs. Lastly, we hypothesise that inhibitory control and spatial working memory performance will mediate the relationship between sleep parameters and increases in PLEs frequency.

## Method

### Participants

The data used in the present study are derived from the CoVenture sample, a 5-year population-based longitudinal cohort study. A total of 3779 high schools students were assessed on a yearly basis from grades 7 to 11 with data collection starting in September 2012 and ending in 2018. Participants were recruited from 31 schools across the greater Montreal area making the cohort epidemiologically representative of the average socioeconomic index of the city’s school districts. After data quality control, a total of 3517 participants who had complete sleep, cognitive and PLEs data for at least two consecutive time points were included in the analysis.

A comprehensive description of the CoVenture Project’s protocol can be found at (ClinicalTrials.gov identifier: NCT01655615)^30^. During the study, 617 out of 3801 participants underwent 3-h long cognitive behavioural therapy sessions. These sessions were specifically designed to prevent drug and alcohol misuse by targeting personality risk factors. However, due to the ongoing experimental blinding, we cannot yet determine the exact effect of these sessions on the study’s main secondary outcomes, such as sleep, cognition, or PLEs. Nevertheless, it’s unlikely that these interventions significantly skewed our results. The group that received the therapy was relatively small, and there is no evidence suggesting that the therapy impacted sleep, cognition, or PLEs. Ethical approval was obtained from the CHU Sainte-Justine Research Ethics Committee in Montreal. All participants actively assented to participate while their parent consented to the study procedures.

### Measures

#### Sleep parameters

Sleep was assessed using a 1 month retrospective questionnaire composed of the following items: sleep quality (SQ: 4=very well, 3=well, 2=badly, 1=very badly), sleep latency (SL: 4=less than 15 min, 3=15 to 30 min, 2=30 to 60 min, 1=more than 60 min) and wake after sleep onset frequency (WASOF: 4=none in the last month, 3=less than once per week, 2=1 to 2 times per week, 1=3 times or more per week). A composite sleep score (CSS) was created by a simple addition of the aforementioned questions’ scores. Sleep quality, wake after sleep onset and sleep latency are the most frequently reported sleep complaints in subjective assessments of patients with schizophrenia^31^ and are more elevated in such patients compared to individuals with other psychopathologies such as bipolar disorder^32^. The sensitivity of self-report questionnaires (73–97.7%) is comparable to that of objective measures derived from hardware devices such as actigraphy and polysomnography (88–98%)^33^. However, the specificity of subjective measures (50–96%) compares favourably to that of objective hardware-based assessments (20–52%)^33^.

#### Psychotic-like experiences

PLEs such as hallucinations, delusional beliefs, suspiciousness, strange experiences, and feelings of grandiosity in the past 12 months were assessed with the PLEs questionnaire for children (PLEQ-C)^34^. The PLEQ-C is a nine-item questionnaire, five of which were adapted from the Diagnostic Interview Schedule. Participants were asked to rate their response on a 3-point scale (0 = not true; 1 = somewhat true; 2 = certainly true). Cronbach’s α ranged from 0.77 to 0.80 between the baseline and fifth assessment. Three items presented positive predictive power (ranging from 71.4 to 100%) for interview-verifiable PLEs^35^. PLEs were present in 13% of children at the age of 11, decreasing progressively over time and reaching 5% by 16 years old^29^. The dimensional measure of PLEs consisted in the sum of all items on the PLEQ-C.

#### Passive avoidance learning paradigm

The passive avoidance learning paradigm^36^ is a modified version of the go/no-go task in which participants are presented with a series of numbers and learn through trial and error which are ‘go’ and which are ‘no-go’ signals. The task had two reinforcement conditions, reward-reward and reward-punishment, but there were no a priori hypotheses about differences in response inhibition errors across these conditions in relation to sleep or psychosis. The variable used in our analyses was the number of commission errors^37^. This variable was chosen over measures reaction time because it is considered a more direct measure of inhibitory control and behavioural outcomes, whereas reaction time may result from a variety of neural mechanisms making it harder to interpret^38^.

#### Spatial working memory

In the find the phone task^39^, participants need to examine several telephones displayed on the screen, touching each to simulate picking up and then hanging up the phone. Our specific version involves identifying a ringing telephone among those shown on the computer screen, while also keeping track of which phones have already been answered to prevent repeated selection. The task involves a set number of trials equal to the number of boxes presented (e.g., four boxes mean four trials). With each new set, the number of boxes increases incrementally from four to eight. The frequency with which participants revisit telephones they have already picked up serves as an indicator of working memory challenges.

#### Culture figure task

The perceptual reasoning of participants was assessed using a selection of nine items derived from Cattell’s Culture Fair Intelligence Test^40^. Performance on these nine items has been shown to be a good approximation of performance on the Raven’s 60-item perceptual reasoning matrices. The items were presented to participants in increasing order of difficulty. The variable used in our analyses is the number of correct answers adjusted for time.

#### Child memory scales ‘dot location’ test

The Wechsler Child Memory Scale^41^, a digital dot location test, was used to assess delayed recall abilities. Participants had to remember the position of 8 dots presented over the course of three trials. An additional (unscored) distractor trial was presented. Participants had to reproduce the initial array of dots immediately and 30 min afterward. The variable used in our analyses is a score based on participants’ ability to reproduce the first three trial sequences after a 30 min delay.

### Analytical approach

Four separate multi-level models were applied to assess the role of distinct cognitive performance measures on the relationship between sleep parameters and PLEs. The mediators are the four cognitive variables the PALP, SWM, CMS and CFT tasks. The model includes random intercepts and slopes at the individual level and controls for sex at the between-group level. Independent variables were person-mean centred. The measurement time points were coded as a wave and included in the within-person concurrent and lagged portions of the analyses.

The models estimated intercept and time parameters and evaluated the contribution of the mean sleep, cognitive and PLEs parameters throughout the 5 years (between-group associations). Changes in the mean value of a parameter for a given participant were compared to changes in related parameters that same year (within-person concurrent association) and the following year (within-person lagged association). The significant within-person concurrent and lagged associations were used to construct and test the significance of mediated paths. No mediation analyses were performed on between-groups associations as the focus of these analyses was to test within-person temporal precedence and mediation across time. A Bayesian estimator was used to manage missing data. All aforementioned analyses were performed using Mplus 8.3 software.

## Results

The sample used in this study consisted of 3517 adolescents (49.5% female, mean age = 12.8, SD = 0.45 years), followed from grades 7–11. A total of 262 (6.9%) participants were excluded from the analysis on the basis that they had not completed a baseline assessment or having completed fewer than two consecutive assessments. The sample’s demographic data, the frequency of PLEs and the CSS are displayed in Table 1.

**Table 1:** Basic descriptive statistics

Demographics	At recruitment	Age		Female		Male	
		12.8 ± 0.45		1864 (49.5%)		1917 (50.9%)	
	Personal and Family Background	Region of birth		Mother’s region of birth		Father’s region of birth	
	Canadian or American	3304		2291		2416	
	European	90		260		237	
	African	65		181		164	
	Caribbean	39		128		115	
	East Asian	104		215		251	
	South Asian	24		145		136	
	Middle Eastern	32		136		91	
	South or Center American	60		146		151	
	Other	38		121		101	
	Don’t know	23		156		117	
Psychotic-Like Experiences	Frequency Score	Year 1	Year 2	Year 3	Year 4		Year 5
	0	675 (31%)	996 (38%)	1123 (41%)	1326 (48%)		1337 (52%)
	1–2	709 (33%)	855 (32%)	967 (35%)	907 (33%)		804 (31%)
	3–4	359 (17%)	386 (15%)	359 (13%)	292 (10%)		231 (9%)
	5–6	178 (8%)	195 (7%)	126 (5%)	104 (4%)		80 (3%)
	7–8	149 (7%)	142 (5%)	122 (4%)	99 (4%)		80 (3%)
	9–10	60 (3%)	34 (1%)	36 (1%)	23 (1%)		18 (1%)
	11–12	25 (1%)	17 (1%)	13 (0%)	16 (1%)		9 (0%)
	13–14	17 (1%)	11 (0%)	19 (1%)	15 (1%)		15 (1%)
	Total	2172	2636	2765	2782		2574
Composite Sleep Score	Average Score	Year 1	Year 2	Year 3	Year 4		Year 5
	4–3.1	85 (4%)	108 (4%)	145 (5%)	184 (7%)		208 (8%)
	3–2.1	333 (15%)	524 (20%)	655 (24%)	720 (26%)		784 (31%)
	2–1.1	118 (55%)	1405 (54%)	1470 (54%)	1481 (54%)		1238 (48%)
	1–0	551 (25%)	555 (21%)	473 (17%)	366 (13%)		324 (13%)
	Total	2154	2592	2743	2751		2554

First, analysis revealed a direct association of poorer CSS with a higher frequency of PLEs at the concurrent within-person level.

For the PALP, analysis revealed a significant within-person association of poorer CSS on higher rates of commission errors both at the concurrent- and lagged-levels. In addition, our analysis revealed a concurrent association of higher rates of commission errors with a higher frequency of PLEs. Based on the aforementioned associations, we performed a mediated path analysis, which resulted in a 1-year lagged L-shaped path. This analysis revealed that increased rates of commission errors on a given year mediated the relationship between poor CSS the previous year and increased PLEs that same year.

Our analysis also revealed significant associations between poorer spatial working memory and increased PLEs at the within-person concurrent and lagged levels. However, no association was found between spatial working memory and CSS at the concurrent or lagged within-person levels.

Lastly, we found between-groups associations of poor CSS with poorer working memory, poorer perceptual reasoning and increased PLEs. We also found between-group associations of increased PLEs with poorer response inhibition, poorer spatial working memory and poorer perceptual reasoning.

**Fig. 1:**
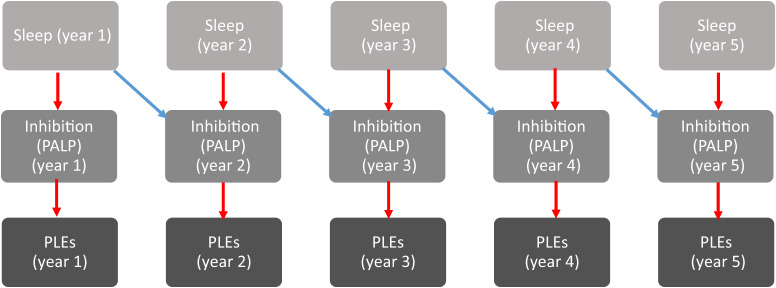
Statistically significant within-person relationships between sleep disruptions and psychotic-like experiences via inhibitory performance. **A** Red arrows represent within-person concurrent associations. **B** Blue arrows represent within-person lagged associations.

**Fig. 2:**
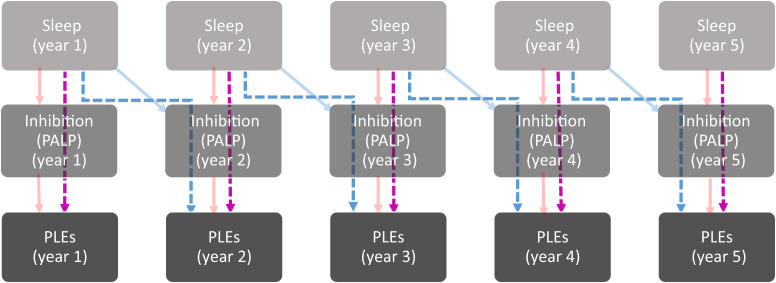
Statistically significant within-person mediated relationships of sleep disruptions with psychotic-like experiences via inhibitory performance. **A** Dashed violet arrows represent the within-person concurrent relationships. **B** Dashed blue arrows represent the within-person L-shaped path relationships. **C** Pale red arrows represent within-person concurrent associations. **D** Pale blue arrows represent within-person lagged associations.

All of the aforementioned results pertaining to direct associations are displayed in Table 2 with the exception of direct and mediated associations between CSS and PLEs, which are displayed in Table 3. The direct and mediated associations involving inhibition are displayed in Figs. 1 and 2 respectively.

**Table 2:** Direct associations

Response Inhibition			
Passive Avoidance Learning Paradigm	Between-Groups Level	Within-Person Concurrent Level	Within-Person Lagged Level
Sleep ON Inhibition	*B* = 0.014, SD = 0.008, *p* = 0.065	*B* = −0.011, SD = 0.004, *p* < 0.001^*^	*B* = −0.013, SD = 0.005, *p* < 0.001^*^
Inhibition ON PLEs	*B* = 1.186, SD = 0.469, *p* = 0.005^*^	*B* = 0.411, SD = 0.170, *p* = 0.005^*^	*B* = −0.093, SD = 0.071, *p* = 0.115

Spatial Working Memory			
Find the phone task	Between-Groups Level	Within-Person Concurrent Level	Within-Person Lagged Level
Sleep ON Working Memory	*B* = 0.103, SD = 0.040, *p* = 0.007^*^	*B* = −0.019, SD = 0.021, *p* = 0.177	*B* = −0.004, SD = 0.030, *p* = 0.458
Working Memory ON PLEs	*B* = 0.084, SD = 0.040, *p* = 0.012^*^	*B* = −0.046, SD = 0.018, *p* = 0.005^*^	*B* = −0.051, SD = 0.023, *p* = 0.010^*^

Perceptual Reasoning			
Cultural Figure Task	Between-Groups Effect	Within-Person Concurrent Level	Within-Person Lagged Level
Sleep ON perceptual reasoning	*B* = 1.538, SD = 0.456, *p* < 0.001^*^	*B* = −0.281, SD = 0.166, *p* = 0.055	*B* = −0.134, SD = 0.220, *p* = 0.265
Perceptual reasoning ON PLEs	*B* = 0.022, SD = 0.007, *p* < 0.001^*^	*B* = 0.000, SD = 0.003, *p* = 0.435	*B* = 0.001, SD = 0.003, *p* = 0.375

Delayed Recall			
Child Memory Scales ‘Dot Location’ test	Between-Groups Level	Within-Person Concurrent Level	Within-Person Lagged Level
Sleep ON Delayed Recall	*B* = 14.096, SD = 23.268, *p* = 0.270	*B* = 8.060, SD = 8.973, *p* = 0.218	*B* = −5.078, SD = 15.138, *p* = 0.360
Delayed Recall ON PLEs	*B* = 0.000, SD = 0.000, *p* = 0.310	*B* = 0.000, SD = 0.000, *p* = 0.280	*B* = 0.000, SD = 0.000, *p* = 0.447

*represents statistically significant findings.

**Table 3:** Sleep to PLEs—direct and indirect associations

Sleep on PLEs paths	Between-Groups Effect	Within-Person Concurrent Level	Within-Person Lagged Level
Sleep ON PLEs	*B* = −1.063, SD = 0.122, *p* = 0.001^*^	*B* = −0.115, SD = 0.047, *p* = 0.005^*^	*B* = −0.052, SD = 0.059, *p* = 0.215
Sleep ON PLEs via commission errors (2 years lagged effect)	*B* = −0.005, SD = 0.003, *p* = 0.005^*^		

*represents statistically significant findings.

## Discussion

This study utilised a longitudinal observational protocol to examine the role of cognition as a mediator between sleep disruptions and PLEs in adolescents. Our results indicate that poorer sleep is associated with concurrent and 1 year lagged disruptions of response inhibition faculties, but not with spatial working memory performance. In addition, poorer performance on both inhibitory and spatial working memory tasks is associated with a concurrent increase in PLEs. A lagged association was also found between spatial working memory and PLEs. The lagged association of sleep with inhibitory deficits is itself associated with an increase in PLEs, forming a 1-year ‘L shaped’ mediatory path. Poorer sleep and PLEs did not appear associated with deficits in delayed recall and perceptual reasoning.

### Sleep and psychotic-like experiences

Our study adds to a growing literature showing an association between sleep disruptions and psychosis. The concurrent association we identified is in line with the results of other studies, which demonstrated a reduction of paranoia and hallucinations among individuals at high risk of psychosis following a course of cognitive behavioural therapy for insomnia (CBTi)^42^. The absence of a lagged association between sleep parameters and PLEs suggests that disrupted sleep unto itself does not lead to long-lasting increases in PLEs. This implies that improvements in sleep may lead to a normalisation of the processes underpinning the risk of psychosis. It should be noted that whereas our questionnaires only capture a single month of sleep disturbances, it is possible that the effects of chronic disruptions are more profound and enduring.

### Relationship of sleep to inhibitory control

Inhibition is the only cognitive domain we measured that showed a significant association with poor sleep. This association was detected both at the concurrent- and lagged-level, suggesting that poor sleep is associated with lasting impairments in inhibition. It is therefore possible that inadequate sleep may impair the neurodevelopmental trajectory of inhibition by interfering with the fine-tuning of underlying inhibitory control processes known to undergo substantial development over the course of adolescence. These findings echo results from healthy participant populations showing a disruption of response inhibition^43^ and increased number of commission errors in emotional go/no-go tasks^44^ following sleep deprivation. This association has far-reaching implications given the important role of inhibition in a wide range of life outcomes such as academic performance, social inclusion and the risk of addiction to drugs and alcohol^25^.

### Inhibition and psychotic-like experiences

The concurrent association between inhibition and PLEs suggests a co-occurring association between these two variables. This is supported by the Waters and al. model of psychosis according to which auditory verbal hallucinations (AVH) result from the combination of inhibitory and self-monitoring deficits^7^. Of note, self-monitoring deficits have been noted in a sub-sample of the CoVenture cohort experiencing high PLEs frequency^45^. These findings also echo previous results in schizophrenia patients who display a higher frequency of go/no-go commission errors, which is in turn associated with a worse total-PANSS and general psychopathology scores^5^. While some studies have failed to show such response inhibition deficits in this patient population, this seems to be attributable to the level of complexity of the task with assessments involving a learning or reward component resulting in worse performance relative to controls^5^. Taken together, these findings suggest that inhibitory deficits may be more central to the aetiology of the disease than previously thought. Two potentiation explanations for this role are that wilful inhibition helps resist the capture of attention by aberrantly salient information or that a form of inhibitory gating prevents such information from becoming salient in the first place.

### Mediation via inhibition

The results of our mediation analysis suggests that disrupted sleep interferes with the development of the inhibitory faculties required to shield awareness against aberrant thoughts and perceptions. While we did not find a direct lagged effect of sleep on PLEs, sleep does appear to have a lasting effect on PLEs through an indirect long-term (lagged) effect on the development of inhibitory control functions during adolescence. According to the extant literature, all three variables appear to share common underlying biological mechanisms, which could be the basis for further research. These include imbalances of the dopaminergic system and a decreased ability of the thalamus to orchestrate the task-appropriate activation of the large-scale networks^46,47^. Beyond their association with PLEs, thalamic abnormalities are associated with sleep disruptions in adolescents at ultra-high risk for psychosis^48^. Taken together, these results indicate that reduced inhibitory control at the very least represents a marker of increasing PLEs. These results indicate that reduced inhibitory control may be considered a direct causal factor of psychosis as suggested by the Waters model of psychosis^7,49^, while variables associated with a deterioration of inhibition (e.g. sleep disruptions) may represent indirect causal factors.

While other studies of sleep disruptions have reported disruptions across a broader range of cognitive domains than those observed in our study, these studies were typically conducted in the context of acute, as opposed to chronic, sleep deprivation. They did not use our unique longitudinal multi-level model, which dissociates between-groups associations (reflecting common vulnerability) from within-person concurrent and lagged associations.

### Working memory and psychosis

The association between working memory and PLEs outlined in our results echoes a scientific consensus supporting a robust deterioration of working memory in individuals with psychosis^50^. The presence of both within-person concurrent and lagged associations suggests working memory may be strongly associated with the development of psychosis. The lagged association in particular suggests that working memory deficits could herald future increases in psychosis risk. Indeed, a number of studies suggest that worse working memory performance represents an additional predictor of first episode psychosis in at risk individuals^8,9^. Other studies suggest that working memory deficits predict the occurrence of AVH^17^, worse functional outcome following first episode psychosis^15^ and negative symptoms severity^18^. While other memory systems are disrupted in psychosis, some studies suggest working memory deficits appear earlier in the developmental phase of the disease, being found as soon as the early prodromal stage, while deficits in long-term recall appear in the late prodromal stage accompanying the beginning of conversion to psychosis^19–21^. A sharp decline in performance across multiple cognitive domains is typical of first episode psychosis^18^.

### Working memory and sleep

While the lack of association between sleep and spatial working memory was surprising, it may be explained by the activation of compensatory neural mechanisms, which appear to help adolescents maintain task performance following sleep restriction^51^. This suggests that working memory deficits found in individuals with a high frequency of PLEs may stem from shared genetic factors found within this population or from unobserved environmental insults.

### Between effects

Our findings also showed between-group associations between inhibition and PLEs, working memory and poor sleep, working memory and PLEs, perceptual reasoning and sleep, perceptual reasoning and PLEs as well as sleep and PLEs. These could be accounted for by shared unobserved variables making their interpretations more speculative. One possibility is the role ascribed to substance use in relation to both adolescent sleep^52^ and cognition^53,54^. For instance, the association between working memory and sleep could be the result of cannabis consumption^55,56^. The fact that our lagged within-effect mediation was tested over and above the observed between-group effects suggests that they are not accounted for by common vulnerability across a large set of covariates shared between cognition, sleep and PLEs.

### Strength and limitations

The main strengths of our study are its longitudinal design and large epidemiologically representative sample. In combination with our multi-level statistical approach, this allows us to observe complex cascades of developmental effects, which unfold from year to year over the course of adolescence. This is important given that the vast majority of studies using neurocognitive assessment are performed on comparatively small samples. The inclusion of random intercepts allows us to control for potential unobserved confounding factors.

The first limitation of our study is that lack of objective sleep measures such as actigraphy and of more comprehensive sleep questionnaires such as the Pittsburgh Sleep Quality Index^57^ or the Consensus Sleep Diary^58^. Instead, our composite score is a general assessment of some of the most frequent sleep complaints reported in the population. This prevents us from studying the contribution of specific dimensions of sleep and makes it more challenging to measure the exact magnitude of the sleep problems afflicting our participants. While the lack of objective measures does limit our ability to assess the association of specific patterns and phases of sleep with our results, it does not compromise their validity. In fact, some studies show that subjectively perceived sleep disruptions are sometimes more predictive of cognitive impairments than objective measures^59^.

The second limitation is that our CSS retrospectively samples only a single month of sleep disruptions per year. This sample may not be entirely representative of the overall sleep quality experienced by participants for the remainder of that year. It is also possible that longer periods of sleep deprivation have longer lasting effects across a broader range of cognitive domains. Future studies are warranted to measure the impact of longer periods of sleep disruption on the development of cognition as it pertains to mental health. An additional confound arises from the mismatch between period covered by the retrospective assessment of the CSS relative to that the PLEQ-C (1 year). This implies that some of the PLEs reported by a participant may have occurred in the weeks following the previous year’s sleep assessment, making it more difficult to determine the exact temporal duration represented by our lagged associations. This concern is partially mitigated by the meditation via inhibitory performance, which happens at the tail end the periods covered by both retrospective assessments.

Finally, given the association of PLEs with general psychopathology^60^ and of cognitive dysfunctions with a wide range of psychopathologies^61^, it is possible that our results reflect a more general effect of such cognitive measures on mental health. Future studies should therefore investigate whether this is the case. If so the inhibition mediated paths we uncovered may increase the overall risk of psychopathology, while the specific diagnosis will be determined by other vulnerabilities (e.g., a higher polygenic risk score).

While more research is needed, our results suggest that targeting cognitive deficits may potentiate existing psychosis prevention interventions, such as cognitive behavioural therapy for psychosis (CBTp). This approach could include cognitive remediation strategies as recommended by numerous clinical international guidelines as it showed promising results in children and adolescents with psychotic symptoms^62^. Elements of CBTi could also be used to augment CBTp. In addition, public health interventions should be aimed at environmental factors known to impact the quality of sleep such as cannabis and social media use^63,64^. In closing, these findings should be taken into account within the ongoing debate surrounding school start times for middle and high school students. While previous research has demonstrated the advantages of a later start time on physical health and academic performance^65^, the cognitive and mental health benefits emphasised in our study underscore that adequate sleep serves as a comprehensive preventive measure across multiple domains of adolescent development.

## Conclusion

This study showed that inhibition mediates the relationship between sleep disruptions and increases in PLEs in adolescents. It also showed a concurrent and lagged association between working memory deficits and increases in PLEs. Lastly, this study showed concurrent and lagged associations between sleep disruptions and increases in PLEs, which is mediated by disrupted response inhibition.

## Data Availability

The data supporting the findings of this study are available upon request from the principal investigator Patricia Conrod. The data are not publicly available due to the nature of the consent.
